# Hyperuricemia might be an early manifestation of undiagnosed adult leukemia in a population-based cohort study

**DOI:** 10.37796/2211-8039.1004

**Published:** 2020-03-28

**Authors:** Shih-Wei Lai, Cheng-Li Lin, Kuan-Fu Liao

**Affiliations:** a College of Medicine, China Medical University, Taichung, Taiwan; b Department of Family Medicine, China Medical University Hospital, Taichung, Taiwan; c Management Office for Health Data, China Medical University Hospital, Taichung, Taiwan; d College of Medicine, Tzu Chi University, Hualien, Taiwan; e Division of Hepatogastroenterology, Department of Internal Medicine, Taichung Tzu Chi General Hospital, Taichung, Taiwan

**Keywords:** adult, hyperuricemia, leukemia, Taiwan National Health Insurance Program

## Abstract

**Background/objective:**

No published population-based study investigates the association between hyperuricemia and undiagnosed adult leukemia in Taiwan. The aim of the study was to investigate whether hyperuricemia might be an early manifestation of undiagnosed adult leukemia in Taiwan.

**Methods:**

A population-based cohort study was conducted to analyze the database of the Taiwan National Health Insurance Program. There were 47708 subjects aged 20 to 84 years with newly diagnosed hyperuricemia as the hyperuricemia group from 2000 to 2013, and 190832 randomly selected subjects without hyperuricemia as the non-hyperuricemia group. The hyperuricemia group and the non-hyperuricemia group were followed for one year to estimate the incidence of new diagnosis of leukemia.

**Results:**

The overall incidence of leukemia was 1.32-fold higher in the hyperuricemia group than the non-hyperuricemia group (0.74 versus 0.55 per 10000 person-months, 95% confidence interval 1.28-1.37). The incidence rate ratio of leukemia was statistically higher in the first 3 months of hyperuricemia diagnosis (incidence rate ratio 4.05).

**Conclusion:**

Adults with hyperuricemia have a higher incidence of being diagnosed with leukemia than those without hyperuricemia. Hyperuricemia might be an early manifestation of undiagnosed adult leukemia. Clinicians should check the complete blood count with differential to detect the possibility of leukemia when adults present with hyperuricemia.

## 1. Introduction

Leukemia is a cancer of blood, arising from blood cell types. Leukemia was as the ninth commonest cancer in the world in 2016 [1]. There were about 467000 new cases of leukemia, which accounted for 2.71% of 17228000 new cancer cases in the world in 2016 [1]. Leukemia ranked the thirteenth leading cause of cancer death in Taiwan in 2018 [2]. There were 1096 deaths due to leukemia, which accounted for 2.25% of 48784 cancer deaths in Taiwan in 2018 [2].

Early manifestations of leukemia before diagnosis might be vague or non-specific, including fatigue, fever, pallor, bleeding tendency, body weight loss, night sweats, insomnia, loss of appetite, ophthalmic involvement, gingival enlargement, and others [3-13]. When leukemia is treated with chemotherapy, hyperuricemia might develop due to rapid turnover of tumor cells [14-25]. Yet, no systemic analysis focuses on the association between hyperuricemia and undiagnosed adult leukemia.

The prevalence of hyperuricemia was high in the general population [26-31], but little is known regarding the association hyperuricemia and undiagnosed adult leukemia in Taiwan. If hyperuricemia is an early manifestation of undiagnosed adult leukemia, these undiagnosed leukemia patients might have a chance to be early detected and they might have a chance to be early treated. Therefore, a population-based cohort study was conducted to investigate this issue.

## 2. Materials and methods

### 2.1. Study design and data source

A population-based cohort study was conducted to analyze the database of the Taiwan National Health Insurance Program. The program was launched in March 1, 1995, and it has covered about 99.7% of 23 million people living in Taiwan [32, 33]. The database included sex, date of birth, and information on ambulatory care, hospitalization care, dental care, emergency care, and prescribed medications [34-40].

### 2.2. Selection of subjects

Subjects aged 20 to 84 years with newly diagnosed hyperuricemia and/or with a new attack of gout from 2000 to 2013 were selected as the hyperuricemia group (the International Classification of Diseases 9th Revision, ICD-9 codes 790.6 and 274). For each subject in the hyperuricemia group, approximately 4 subjects without hyperuricemia and gout were randomly selected as the non-hyperuricemia group. The index date was defined as the date of subjects being diagnosed with hyperuricemia or gout. The hyperuricemia group and the non-hyperuricemia group were matched with sex, age (every 5-year interval), and the year of index date. Subjects with a history of any cancer before the index date were excluded from the study.

### 2.3. Major outcome

The major outcome was a new diagnosis of leukemia (ICD-9 codes 204-208) during the follow-up period. All study subjects were followed for one year.

### 2.4. Statistical analysis

We compared the differences of sex and age between the hyperuricemia group and the non-hyperuricemia group by the Chi-square test for categorized variables and the *t*-test for continuous variables. The incidence of leukemia was estimated as the event number of leukemia detected during the follow-up period, divided by the total follow-up person-months for each group. The incidences of leukemia stratified by sex, age, and follow-up period between the hyperuricemia group and the non-hyperuricemia group were estimated. The incidence rate ratio (IRR) and the 95% confidence interval (CI) of leukemia in the hyperuricemia group versus the non-hyperuricemia group were estimated. All analyses were performed by the SAS 9.2 version (SAS Institute Inc., Carey, North Carolina, USA). The results were considered statistically significant when two-tailed *P* values were <0.05.

## 3. Results

### 3.1. Baseline information of the study population

Table 1 reveals the baseline information of the study population. There were 47708 subjects in the hyperuricemia group and 190832 subjects in the non-hyperuricemia group. The mean ages (standard deviation) of the study subjects were 48.6 (15.5) years for the hyperuricemia group and 48.6 (15.4) years for the non-hyperuricemia group, without statistical significance (*t*-test, *P* = 0.27).

### 3.2. Incidence of leukemia stratified by sex age, and follow-up period

Table 2 reveals that after adjusted for sex and age, the overall incidence of leukemia was 1.32-fold higher in the hyperuricemia group than that in the non-hyperuricemia group (0.74 versus 0.55 per 10000 person-months, 95% CI 1.28-1.37). The incidences of leukemia, as stratified by sex, age, and follow-up period, were all statistically higher in the hyperuricemia group than those in the non-hyperuricemia group. Subjects aged 65 to 84 years in the hyperuricemia group had a particularly higher incidence of leukemia (1.5 per 10000 person-months). The incidence rate ratio of leukemia was statistically higher in the first 3 months of follow-up (incidence 0.28 versus 0.07 per 10000 person-months, incidence rate ratio 4.05, 95% CI 3.90-4.20). The incidence rate ratio was reduced to 1.23 during 3-12 months of follow-up (incidence 0.89 versus 0.72 per 10000 person-months, 95% CI 1.19-1.28).

## 4. Discussion

To the best of our knowledge, this is the first population-based study to investigate the association between hyperuricemia and undiagnosed adult leukemia in Taiwan. No other study can be compared with each other. In order to reduce the biased results in our study, subjects with a history of any cancer before the index date were excluded from the study. All study subjects were followed until leukemia was newly diagnosed or for one year. Therefore, leukemia patients in our study were new cases. The diagnosis of hyperuricemia in the study was really prior to the confirmed diagnosis of leukemia. Therefore, hyperuricemia is less likely to be secondary to chemotherapy for leukemia or other cancers.

Although not unexpected findings, we noted that the overall incidence of leukemia was 1.32-fold higher in the hyperuricemia group than that in the non-hyperuricemia group. The incidence rate ratio of leukemia was statistically higher in the first 3 months (incidence rate ratio 4.05). Although the outcome number was small (only 42 leukemia patients), our findings highlight that hyperuricemia might be an early manifestation of undiagnosed adult leukemia, particularly within three months of hyperuricemia diagnosis.

We reviewed the relevant literature to explain the association between hyperuricemia and undiagnosed adult leukemia. Leukemia cells have a tendency of rapid turnover, which leads to rapid breakdown of these tumor cells and subsequent hyperuricemia [14-17, 22]. Because early manifestations of leukemia before confirmed diagnosis might be vague or non-specific, it is difficult to early diagnose leukemia. We suggest that clinicians should check the complete blood count with differential to detect the possibility of leukemia when adults present with hyperuricemia.

## 5. Limitation

Several limitations of the study should be discussed. First, due to the inherent limitation of the database, other manifestations of leukemia before confirmed diagnosis were not documented. We could not determine hyperuricemia or other manifestations caming first. During disease progression of different stages, leukemia patients would have different manifestations, but our study could not verify it. Second, due to the eligible number of leukemia patients being small, we could not make a comparative analysis between acute and chronic leukemia. We could only analyze the association between hyperuricemia and the overall leukemia. Moreover, it indicates a future research direction. Third, In view of the availability of the Taiwan's medical care, it is not necessary to spend one year to diagnose leukemia since the onset of leukemia-related manifestations. That was why subjects in our study were only followed for one year.

We conclude that adults with hyperuricemia have a higher incidence of being diagnosed with leukemia than those without hyperuricemia. Hyperuricemia might be an early manifestation of undiagnosed adult leukemia. Clinicians should check the complete blood count with differential to detect the possibility of leukemia when adults present with hyperuricemia.

## Figures and Tables

**Table 1:** Baseline characteristics of the study population.

Variable	Non-hyperuricemia N = 190832		Hyperuricemia N = 47708		*P* value^a^
	n	(%)	n	(%)	
Sex					0.001
Female	61014	(32.0)	16154	(33.9)	
Male	129818	(68.0)	31554	(66.1)	
Age group (years)					0.001
20-39	61049	(32.0)	15497	(32.5)	
40-64	97551	(51.1)	23791	(49.9)	
65-84	32232	(16.9)	8420	(17.6)	
Age (years), mean ± standard deviation^b^	48.6 ± 15.4		48.6 ± 15.5		0.27

Data are presented as the number of subjects in each group with percentages given in parentheses.

a Chi-square test and.

b *t*-test comparing the hyperuricemia group and the non-hyperuricemia group.

**Table 2:** Incidence of leukemia stratified by sex, age, and follow-up period between the hyperuricemia group and the non-hyperuricemia group.

Non-hyperuricemia					Hyperuricemia					
Variable	N	Event	Person-months	Incidence^a^	N	Event	Person-months	Incidence^a^	IRR^b^	(95% CI)
All	190832	126	2276984	0.55	47708	42	571180	0.74	1.32	(1.28, 1.37)
Sex										
Female	61014	50	728563	0.69	16154	16	193388	0.83	1.19	(1.12, 1.26)
Male	129818	76	1548421	0.49	31554	26	377791	0.69	1.42	(1.36, 1.48)
Age group (years)										
20–39	61049	21	728860	0.29	15497	10	185809	0.54	1.88	(1.77, 1.99)
40–64	97551	68	1167195	0.58	23791	17	285081	0.60	1.04	(0.98, 1.09)
65-84	32232	37	380929	0.97	8420	15	100291	1.50	1.54	(1.42, 1.66)
Follow-up period (months)										
3	190832	4	571701	0.07	47708	4	143077	0.28	4.05	(3.90, 4.20)
3-12	190306	122	1705283	0.72	47666	38	428103	0.89	1.23	(1.19, 1.28)

a Incidence rate: per 10000 person-months.

b IRR (incidence rate ratio, adjusted for sex and age): hyperuricemia versus non-hyperuricemia (95% confidence interval).
